# Supplementary material for: ProteinShader: illustrative rendering of macromolecules
Source: BMC Struct Biol. 2009 Mar 30;9:19. doi: 10.1186/1472-6807-9-19 (PMC2672931; doi:10.1186/1472-6807-9-19)
Supplement: Additional file 1 — ProteinShader program without source code. This compressed file contains the complete ProteinShader program including associated libraries, but no source code. A README.txt file gives an overview of the ProteinShader distribution, and the index.html file in the help subdirectory has directions on getting started with the program as well as a set of tutorials. [file 1472-6807-9-19-S1.zip › ProteinShader-beta-0_9_4-binary/help/api/org/proteinshader/structure/exceptions/class-use/MissingAATypeException.html]

Uses of Class org.proteinshader.structure.exceptions.MissingAATypeException (ProteinShader API)


|  |  |  |
| --- | --- | --- |
| **Overview** \| **Package** \| **Class** \| **Use** \| **Tree** \| **Deprecated** \| **Index** \| **Help** | |  |
| PREV   NEXT | **FRAMES**    **NO FRAMES**     **All Classes** |  |


---


## **Uses of Class org.proteinshader.structure.exceptions.MissingAATypeException**

| Packages that use MissingAATypeException | |
| --- | --- |
| **org.proteinshader.structure** | Holds the classes that store information from a Protein Data Bank file: Structure, Model, Chain, AminoAcid, Heterogen, Water, Atom, Bond, Helix, BetaStrand, Loop, *etc*. |

Uses of MissingAATypeException in org.proteinshader.structure

| Methods in org.proteinshader.structure that throw MissingAATypeException | |
| --- | --- |
| `AminoAcid` | `Chain.addNewAminoAcid(AminoAcidEnum type, String residueID)`             Creates a new AminoAcid with the type and Residue ID given as arguments, adds the AminoAcid to the Chain's collection of AminoAcids, and returns a reference to the new AminoAcid. |
| `Atom` | `Structure.addNewAtom(int atomSerialNo, AtomEnum atomType, String atomID, String aminoAcidType, String residueID, String chainID, String modelID, double temperature, int charge, double occupancy, String altLocation, double x, double y, double z, VisibilityEnum visibility)`             Creates a new atom with the atomID given as an argument, adds the new Atom to the requested Model-Chain-AminoAcid, and then returns a reference to the new Atom. |

Constructors in org.proteinshader.structure that throw MissingAATypeException

`AminoAcid(AminoAcidEnum type,
String residueID,
String chainID,
String modelID,
String structureID)`
  
          Constructs an AminoAcid with the type and IDs given as arguments.

---


|  |  |  |
| --- | --- | --- |
| **Overview** \| **Package** \| **Class** \| **Use** \| **Tree** \| **Deprecated** \| **Index** \| **Help** | |  |
| PREV   NEXT | **FRAMES**    **NO FRAMES**     **All Classes** |  |


---

# *Copyright © 2007-2008*
